# The reliability of the augmented Lehnert-Schroth and Rigo classification in scoliosis management

**DOI:** 10.4102/sajp.v77i2.1568

**Published:** 2021-11-02

**Authors:** Burçin Akçay, Tuğba Kuru Çolak, Adnan Apti, İlker Çolak, Önder Kızıltaş

**Affiliations:** 1 Department of Physiotherapy and Rehabilitation, Faculty of Healthy Sciences, Bandırma Onyedi Eylül University, Balıkesir, Turkey; 2 Department of Physiotherapy and Rehabilitation, Faculty of Health Sciences, Marmara University, Istanbul, Turkey; 3 Department of Physiotherapy and Rehabilitation, Faculty of Healthy Sciences, İstanbul Kültür University, Istanbul, Turkey; 4 Department of Orthopaedics and Traumatology, Kartal Dr. Lütfi Kırdar Education and Research Hospital, Istanbul, Turkey; 5 Department of Orthotics and prosthetics, Denge Ortopedi, Istanbul, Turkey

**Keywords:** scoliosis, augmented Lehnert-Schroth classification, Rigo classification, conservative treatment, reliability

## Abstract

**Background:**

In pattern-specific scoliosis exercises and bracing, the corrective treatment plan differs according to different curve patterns. There are a limited number of studies investigating the reliability of the commonly used classifications systems.

**Objective:**

To test the reliability of the augmented Lehnert-Schroth (ALS) classification and the Rigo classification.

**Methods:**

X-rays and posterior photographs of 45 patients with scoliosis were sent by the first author to three clinicians twice at 1-week intervals. The clinicians classified images according to the ALS and Rigo classifications, and the data were analysed using SPSS V-16. Intraclass correlation coefficients (ICCs) and standard error measurement (SEM) were calculated to evaluate the inter- and intra-observer reliability.

**Results:**

The inter-observer ICC values were 0.552 (ALS), 0.452 (Rigo) for X-ray images and 0.494 (ALS), 0.518 (Rigo) for the photographs. The average intra-observer ICC value was 0.720 (ALS), 0.581 (Rigo) for the X-ray images and 0.726 (ALS) and 0.467 (Rigo) for the photographs.

**Conclusions:**

The results of our study indicate moderate inter-observer reliability for X-ray images using the ALS classification and clinical photographs using the Rigo classification. Intra-observer reliability was moderate to good for X-ray images and clinical photographs using the ALS classification and poor to moderate for X-ray and clinical photographs using the Rigo classification.

**Clinical implications:**

Pattern classifications assist in creating a plan and indication of correction in specific scoliosis physiotherapy and pattern-specific brace applications and surgical treatment. More sub-types are needed to address the individual patterns of curvature. The optimisation of curve classification will likely reduce failures in diagnosis and treatment.

## Introduction

Scoliosis is a three-dimensional deformity of the spine and trunk, including lateral flexion and rotation of the vertebrae with sagittal plane changes. Although there are many epidemiological causes (such as congenital and neuromuscular), adolescent idiopathic scoliosis (AIS) is one of the main types of scoliosis, with a prevalence of 80% – 90% (Horne, Flanney & Usman 2014; Moramarco et al. 2020; Weiss 2010a; Weiss, Lehner-Schroth & Moramarco 2018).

Theoretically, an unlimited number of curve patterns with different geometrical entities can be seen. Current management strategies for idiopathic scoliosis include observation, physiotherapy that includes scoliosis-specific exercises, bracing, and surgery (Bettany-Saltikov et al. 2014; Horne et al. 2014; Moramarco et al. 2020). However, for ease of treatment approaches and to address the biomechanical properties and planning of correction, several curve classification systems have been developed for exercise, brace, and surgical treatments.

Curve patterns for scoliosis were first identified by Ponseti and Friedmann in 1950 and Moe and Kettleson in 1970 for surgical treatments (Moe & Kettleston 1970; Ponseti & Friedman 1950). In the late 1970s, a simple, functional classification for approaching different curve patterns with physiotherapy was established by Lehnert-Schroth (2007). Chêneau also used this simple classification for the construction of braces (Weiss 2010b). In the 1980s, the King classification was developed to help surgeons approach the curves properly during operations (King, Moe & Winter 1983). Then, the more complex Lenke classification was developed by surgeons because the use of the King classification had led to imbalanced post-surgical results and seemed to lack reliability (Lenke et al. 2001). Rigo implemented a new classification for brace treatment with 15 different curve patterns, derived from the Lenke classification and introduced a new classification in 2010. In the same year, the augmented Lehnert-Schroth (ALS) classification was developed by Weiss (Rigo 2004; Rigo, Villegrasa & Gallo 2010; Weiss 2010a).

The corrective treatment plan for pattern-specific scoliosis exercises and bracing differs according to the different curve patterns. Therefore, these patterns of curve correction need to be based on a standardised classification for all types of professionals engaged in the conservative treatment of scoliosis. There are a limited number of studies that have investigated the reliability of the Rigo classification, and no studies have investigated the reliability of the ALS classification, including comparison of the reliability of the two classifications (Chen & Liao 2019; Rigo et al. 2010). Thus, the purpose of our study was to test the reliability of the ALS classification and the Rigo classification, which are commonly used in clinical practice.

## Method

Our reliability study to determine inter- and intra-observer reliability, X-rays and clinical photographs recruited patients with AIS from the Dr Lütfi Kırdar Kartal Training and Research Hospital, Department of Orthopaedics and Traumatology.

Three experienced clinicians volunteered to participate in our study. The clinicians who undertook the classifications consisted of an orthopaedic surgeon (5 years’ experience), a physiotherapist (13 years’ experience) and an orthotist (11 years’ experience), with clinical and academic experience in scoliosis. A quota sampling method was used to select X-ray images and clinical photographs of the patients.

X-ray images and clinical photographs of 45 AIS patients admitted to our department between January and February 2020 were collected. Inclusion criteria of the patients were as follows: having been diagnosed with idiopathic scoliosis between the ages of 10 and 16 years, suitable for brace use and a Cobb angle between 20º and 50º. Exclusion criteria of the patients were as follows: non-idiopathic scoliosis, any rheumatologic or orthopaedic disease, any mental disorder, or a history of spinal operation.

### Procedure

Inter-rater reliability was undertaken to establish the degree of agreement among the three independent observers and intra-rater reliability was undertaken to establish the degree of agreement of repeated observations of a single rater. The clinicians rated all the X-ray images and photographs twice to determine the inter- and intra-rater reliability of the ALS and Rigo classifications.

Anteroposterior X-ray images and pictures were gathered from each of the participants’ medical records. The first author listed the X-ray images and clinical photographs of 45 AIS patients in a mixed order and e-mailed them to the clinicians as two separate files. The clinicians classified the X-ray images and clinical photographs according to the ALS and Rigo classifications and then sent them to the first author. All clinicians completed the first classification section. A week later, the first author changed the order of the X-ray images and photographs, renumbered and resent them to the clinicians. The clinicians again classified the X-ray images and clinical photographs in the files. This was the second classification section. Thus, the same X-ray images and clinical photographs were classified according to two different classification systems at two different times by the same clinicians under the same conditions.

### Classification methods

The ALS classification was developed as an expansion of the Lehnert-Schroth classification and included seven different curvature types. The first type is 3CH (3 curve hip) and it means functional 3-curve with hip prominence. The second type is 3CTL (3 curve thoracolumbar) and it means functional 3-curve with hip prominence thoracolumbar. Third type is 3C (3 curve) and it means functional 3-curve balanced. Fourth type is 3CL (3 curve lumber) and it means functional 3-curve with a long lumbar counter curve. Fifth type is 4C (4 curve) and it means functional 4-curve double. Sixth type is 4CL (4 curve lumbar) and it means functional 4-curve with major lumbar curvature. Seventh type is 4CTL (4 curve thoracolumbar) and it means functional 4-curve with major thoracolumbar curvature (Weiss 2010a, 2010b; Weiss et al. 2018).

Rigo implemented a new classification for brace treatment with 15 different curve patterns, derived from the Lenke classification in 2004 (Rigo 2004). Later in 2010, the classification was modified to define specific correction principles when treating with a particular type of brace. The Rigo classification includes four basic curve types: (1) three curves, (2) four curves, (3) non three, non four and (4) single lumbar or thoracolumbar. The three curves basic type is divided into sub-types A1 (single long thoracic or partial lumbar curve), A2 (single thoracic or minimally to non functional lumbar), and A3 (single major thoracic or minor lumbar curve) concerning lumbar configuration. The four curves basic types are divided into sub-types B1 (double thoracic and lumbar or thoracic and thoracolumbar curve) and B2 (major thoracolumbar curve with minor thoracic curve) concerning thoracic configuration. Non three, non four basic type is divided into sub-types C1 (single thoracis without lumbar curve) and C2 (thoracic major and lumbar minor or double thoracic and lumbar curve), also concerning lumbar configuration. Single lumbar and thoracolumbar are also called E1 (single lumbar with no thoracic curve) and E2 (single thoracolumbar curve without thoracic curve), respectively. The D modifier defines an upper thoracic structural curve (Rigo et al. 2010).

### Data analysis

Data analysis was performed using SPSS V 16. Intraclass correlation coefficients (ICCs) for the same observer and different observers were calculated with 95% confidence intervals for both classifications. The standard error of measurements (SEMs) were calculated using the formula: SEM = SD√ (1 − ICC). ICC values < 0.5 are indicative of poor reliability, values between 0.5 and 0.75 indicate moderate reliability, values between 0.75 and 0.9 indicate good reliability and values > 0.90 indicate excellent reliability (Koo & Li 2016).

### Ethical considerations

Ethical approval for our study was obtained from the Bandırma Onyedi Eylül University – Ethics Committee of Non-interventional research in Health Sciences and was conducted in compliance with the Helsinki Declaration. Patients and their parents were informed about our study, and written consent was obtained prior to the start of our study.

## Results

The inter-observer ICC value was 0.552 for the evaluation of the reliability of the X-ray images and 0.494 for the clinical photographs using the ALS classification. The inter-observer ICC value was 0.452 for the evaluation of the reliability of the X-ray images and 0.518 for the clinical photographs using the Rigo classification (Table 1).

**TABLE 1:** Inter-observer reliability analysis for augmented Lehnert-Schroth classification and Rigo classifications.

Inter-observer reliability	ALS X-ray image	ALS photograph	Rigo X-ray image	Rigo photograph
ICC	0.552	0.494	0.452	0.518
95% CI	0.384–0.702	0.318–0.657	0.269–0.625	0.345–0.676
SEM	2.87	3.65	4.21	4.76

ALS, augmented Lehnert-Schroth classification; ICC, intraclass correlation coefficients; CI, confidence interval; SEM, standard error of measurements.

The intra-observer ICC values fluctuated from 0.648 to 0.807 (average: 0.720) for the evaluation of the reliability of the X-ray images using the ALS classification and fluctuated from 0.616 to 0.878 (average: 0.726) for the evaluation of the reliability of the clinical photographs using the ALS classification. The intra-observer ICC values fluctuated from 0.446 to 0.670 (average: 0.581) for the evaluation of the reliability of the X-ray images using the Rigo classification and fluctuated from 0.126 to 0.656 (average: 0.467) for the evaluation of the reliability of the clinical photographs using the Rigo classification (Table 2).

**TABLE 2:** Intra-observer reliability reliability analysis for augmented Lehnert-Schroth classification and Rigo classifications.

Variables	ALS X-ray image	ALS photograph	Rigo X-ray image	Rigo photograph
**Rater 1**				
ICC	0.707	0.684	0.629	0.126
95% CI	0.523–0.827	0.492–0.813	0.414–0.778	0.301–0.721
SEM	1.49	2.13	2.65	3.9
**Rater 2**				
ICC	0.807	0.878	0.446	0.656
95% CI	0.675–0.889	0.791–0.932	0.179–0.652	0.480–0.808
SEM	2.0	1.36	3.19	2.94
**Rater 3**				
ICC	0.648	0.616	0.670	0.620
95% CI	0.440–0.790	0.396–0.769	0.468–0.805	-0.213–0.365
SEM	1.79	2.4	2.51	3.14

ALS, augmented Lehnert-Schroth classification; ICC, intraclass correlation coefficients; CI, confidence interval; SEM, standard error of measurements.

## Discussion

The results of our study indicate moderate inter-observer reliability for X-ray images using the ALS classification and clinical photographs using the Rigo classification. Intra-observer reliability was moderate to good for X-ray images and clinical photographs using the ALS classification and poor to moderate for X-ray and clinical photographs using the Rigo classification.

Classification schemes help clinicians organise their thoughts about the type of problem being treated and design appropriate treatment. Therefore, classification systems not only provide an approach to a problem but also suggest a treatment method (Lenke et al. 1998; Smith et al. 2008). The use of a classification system is crucial for brace design and planning an exercise programme. There are many different conservative treatment approaches in treating idiopathic scoliosis, and each approach uses different systems (classifications) to prescribe the corrective braces and exercises (Berdishevsky et al. 2016; Rigo 2004; Rigo et al. 2010; Weiss 2010a).

The ALS classification was developed by Weiss in 2010 in the hope that it would be a valuable and easy-to-use practice-based aid for scoliosis health professionals. The ALS classification includes detailed radiological and clinical definitions. Today this classification is used for the application of Schroth Best Practice (SBP) exercises and the application of the Gensingen brace (GBW) (Horne et al. 2014; Weiss et al. 2018). Our study indicated moderate inter-observer reliability and good intra-observer reliability for X-ray images, and poor inter-observer reliability and good intra-observer reliability for clinical photographs using the ALS classification. These results showed that for the ALS classification, radiological estimation of curve patterns seems more reliable than the clinical estimation. No previous study investigating the validity and reliability of the ALS classification could be found in the literature, so that no comparisons could be made for the results of the ALS classification.

Rigo (2004) derived his first curve classification from the Lenke classification (Horne et al. 2014). In 2010, Rigo reworked the classification and introduced new terminology. The Rigo Radiological Classification System uses objective radiological criteria to confirm the functional curve type to correlate with the Rigo System Chêneau brace design and physiotherapy. Rigo et al. (2010) reported good inter-observer reliability as well as intra-observer reliability regarding the radiological criteria of the Rigo classification. Rigo et al. (2010) reported the intra-observer reliability as a Kappa value of 0.87 and the average inter-observer reliability as a Kappa value of 0.71 (0.61–0.81). Chen and Liao (2019) reported that the mean Kappa value for the inter-observer reliability test was 0.89, and for the intra-observer test, it was 0.91. The inter-observer ICC values were 0.452 and 0.518 (poor and moderate) for the reliability of the evaluation of the X-ray images and for the clinical photographs using the Rigo classification. The intra-observer ICC values fluctuated from poor to moderate for the evaluation of the reliability of the X-ray images and clinical photographs (average: 0.581 and 0.467) using the Rigo classification. These results showed that for the Rigo classification, clinical estimation of curve patterns seems more reliable than the radiological estimation. Thus, the results of previous studies could not be reproduced in respect of the reliability of the Rigo classification. It is surprising that in a more recent article, the complex and comprehensive Rigo classification was presented with even better reliability than in the study by Rigo (2010) himself. Also, compared to previous studies, our study showed the lowest reliability for the Rigo classification (Rigo et al. 2010; Weiss 2010a).

The ALS and Rigo classifications are derived from three and four curve patterns described in the Lehnert-Schroth classification and include sub-types of these curve patterns (Rigo et al. 2010; Weiss 2010a). We showed that for the ALS classification, the radiological estimation of curve patterns seems more reliable than the clinical estimation, but for the Rigo classification, the clinical estimation of curve patterns seems more reliable than the radiological estimation. We also demonstrated higher intra-rater reliability scores for the ALS classification than for the Rigo classification and similar inter-rater reliability for both classifications.

Intraclass correlation coefficients are currently widely used in conservative care medicine to evaluate inter-rater, test–retest and intra-rater reliability and is suitable for studies with two or more raters (Koo & Li 2016). In our study, there were three raters, and the reliability analysis was presented with ICC values. Similarly, in studies by Rigo et al. (2010) and Chen et al. (2019), three observers measured X-rays for inter-observer reliability, and all X-rays were re-measured for intra-observer reliability. It is seen in these studies that the reliability of the classification was made only on X-ray (Chen & Liao 2019; Rigo et al. 2010). The distinguishing feature of our study is that the evaluation was made from clinical photographs of the patients as well as X-rays. During treatment, the pattern of spinal curvatures may change as children continue to grow, so radiological and clinical evaluations are essential in the management of scoliosis (Borysov et al. 2020). In this context, our study emphasises the importance of the classifications to be clinically reliable.

A limitation of our study was that a larger sample group could not be reached because of the coronavirus disease 2019 (COVID-19) pandemic. Our results are important as, to our knowledge, this is the first study that included the reliability analysis of the ALS classification. In addition, the clinical implications of our results using the clinical photography method are also important. The raters in our study were familiar with the classification and terminology and had an average of 9.66 years of experience in conservative scoliosis treatment. There may be differences in decision-making using the classifications among individuals with different levels of experience. Therefore, further studies on this topic are needed from other independent study groups.

## Conclusion

Our results indicate moderate inter-observer reliability for X-ray images using the ALS classification and clinical photographs using the Rigo classification. Intra-observer reliability was moderate to good for Xray images and clinical photographs using the ALS classification and poor to moderate for X-ray and clinical photographs using the Rigo classification. In a scoliosis physiotherapy programme which is pattern-specific, it is necessary to distinguish between certain patterns of curvature. The best possible correction can be achieved with pattern-specific corrective movements, and the increase in any counter curves could also be avoided. Pattern classifications assist in creating a plan and indication of correction not only in specific scoliosis physiotherapy, but also for pattern-specific brace applications and for surgical treatment. To obtain an effective brace treatment, the Lehnert-Schroth classification cannot be regarded as being sufficient. As more sub-types are needed to address the individual patterns of curvature, the ALS and Rigo classifications were established. The optimisation of curve classification will likely reduce failures in diagnosis and treatment.
